# Oral microbiota in oropharyngeal cancers: Friend or foe?

**DOI:** 10.3389/fonc.2022.948068

**Published:** 2022-09-13

**Authors:** Riccardo Nocini, Lorenzo Lo Muzio, Davide Gibellini, Giovanni Malerba, Michele Milella, Salvatore Chirumbolo, Nicoletta Zerman

**Affiliations:** ^1^ Department of Surgery, Dentistry, Paediatrics and Gynaecology, University of Verona, Verona, Italy; ^2^ Department of Clinical and Experimental Medicine, University of Foggia, Foggia, Italy; ^3^ Department of Diagnostic and Public Health, University of Verona, Verona, Italy; ^4^ Department of Neurosciences, Biomedicine and Movement Sciences, University of Verona, Verona, Italy; ^5^ Department of Medicine, University of Verona, Verona, Italy

**Keywords:** oral cancer, oral microbiome dysbiosis, 16sRNA, oral immunity, oropharyngeal cancer (OPSCC)

## Abstract

Oral microbiome is a complex population of micro-organisms, which by cross-talking with the local immune system, plays a major role in the immune homeostasis of the oral cavity, further contributing in the physiology of the gastro-intestinal microbiota. Understanding their involvement in the onset and pathogenesis of oropharyngeal cancers is paramount, despite very few reports deal with the fundamental role exerted by oral microbiota disorders, such as dysbiosis and impairment in the oral microbiome composition as causative factors in the development of oropharyngeal tumors. Current research, *via* metabolomic or meta-transcriptomic analyses, is wondering how this complex microbial population regulates the immune homeostasis in oral and pharyngeal mucosa and whether changes in bacterial composition may give insights on the role of oral microbiome in the development of oropharyngeal tumors, so to prevent their occurrence.

## Introduction

What is generally known in dentistry is that oral hygiene should have a major impact on the prevention of oral diseases, even including cancer, and actually many experts are wondering if oral bacteria can be considered as direct causative agents promoting oropharyngeal tumors (1–4). Moreover, teeth and gums associated pathologies, e.g. periodontitis, seem to be a leading cause of oropharyngeal cancers, although lifestyle and voluptuary habits, associated with age, sex, smoke, tobacco and alcohol abuse, have to be included in the causative group of factors (5).

The complex relationship between oral microbiota and tumor development in the oral-gastro-intestinal tract, was recently reviewed, reporting that a major shift in the composition of oral microbiota between apparently healthy subjects and cancer patients is demonstrable (6). According to Mascitti et al., the most predominant phyla in the majority of cancer patients are *Firmicutes* and *Actinobacteria* (6).

Actually, Schmidt et al., reported that changes in the abundance of *Firmicutes* (genus *Streptococcus*) were associated with oral pre-cancers, suggesting that any oral lesion is related to significant shifts in the oral microbiome and that these changes are associated with the rate of occurrence in oral cancers (7). *Actinobacteria* are particularly represented, alongside with *Firmicutes*, *Bacteroides*, and *Fusobacteria*, in the tongue coating (TC) microbiota (8). The TC microbiota is particularly related with the development of upper gastrointestinal cancers (8) and moreover, in the oral squamous cell carcinoma (OSCC) the Actinobacteria genus *Veillonella* and the species *Granulicatella adiacens Veillonella rogosae* and *Streptococcus sanguinis* are particularly abundant in normal paracancerous tissues of OSCC patients (9).

Searching for envisaging the particular strain or species and/or bacterial composition in the oral microbiome as a biomarker of a possible carcinogenesis, might be a leading target of current oral oncology, despite many controversial aspects are far to be fully elucidated (10). In this context, some authors have introduced the concept of “resilience” to describe the behavior of oral microbiota facing new microenvironmental changes towards the prevention of a shift in the microbial composition, which may lead to immune impairments and cancer onset (6, 7). Briefly speaking, a homeostatic interplay microbiota-oral immunity, might be the actual source of this resilience, i.e. the complex cross-talk of microbes and oral immunity may be the key for maintaining a tolerant, anti-inflammatory phenotype, in the oral micro-environment. In this perspective, the thorough knowledge of the many interrelationships among different bacterial phyla and genera in the community of the oral microbiome, *via* metabolomic or meta-transcriptomic analyses, should give insightful clues about possible existing relationships between oral microbiome dysbiosis and cancer pathogenesis (6, 10, 11).

As a matter of fact, DNA sequencing of the oral microbiota is gaining a growing interest. It relies on a thorough and accurate pick up of the oral microbiome (saliva, teeth, and other microbial niches) and its DNA profiling, then allowing to draw the molecular profiles of the whole set of microorganisms, including such strains or species that do not grow easily in standard culture media.

Genetic profiling can be performed using at least two different approaches: the most common 16S rRNA gene sequencing (16Ss), and the most recent shotgun metagenomic sequencing (SMs).

16Ss is based on the sequencing of one single gene, the 16S rRNA gene (12, 13).

In particular, sequencing enrolls one or more of the 9 hyper-variable regions (V1-V9) of the gene. Each hyper-variable region is supposed to recognize specific microbial taxonomic groups, using proper bioinformatic approaches together with bacterial taxonomic reference databases (14, 15). Although the sequencing of the full 16S rRNA gene would increases the chances to cover in greater detail the microbial taxonomy of a given sample, this is not generally pursued, since the standard approach employs the sequencing of only 2-3 hyper-variable regions. Since different microbial communities colonize different oral environments/niches (lips, cheeks, palate, teeth, gingival sulcus, saliva), a successful identification of bacterial at a good taxonomic depth (genus or rarely species) is not granted when sequencing only a subgroup of the hyper-variable regions or even when sequencing the entire 16S rRNA. Most importantly, it appears clear that the choice of the hyper-variable region undergoing sequencing might dramatically impact on the final overall results (identification and quantification of the microbial taxonomic groups) (16).

SMs involves the sequencing of small random fragments of the microbial DNA to identify the species and genes present in the samples. Unlike 16Ss, SMs reads DNA fragments from all the microbial genomes, rather than just one specific gene region of the 16 rRNA gene. Sequencing can identify and profile bacteria, fungi, viruses and many other types of microorganisms at the same time (17). Moreover, with this approach, which requires more complex bioinformatics methods and is more expensive than 16Ss, researchers can also identify genes (the metagenome), which provide additional information about microbiome functional potential (e.g. virulence, metabolic or antibiotic resistance genes). When it is necessary to look at the species and strains within the microbiome of interest, SMs is therefore more powerful than 16Ss. SMs represents a new incoming approach to study bacterial, fungal and viral microbiomes in terms of composition and functions of the microbial community (18, 19). Moreover, it is likely that future studies will expand the complexity of the association analyses by including interactions between the oral-microbiome and the host genome in order to target the most susceptible oral microbiota- host profiles.

Actually, the major question experts should raise is whether a thorough knowledge of the composition of the oral microbiome *via* genomic and metabolomic analysis may serve as a leading diagnostic tool in forecasting the risk of a cancer onset.

## The oral microbiome composition in the hygiene perspective of oropharyngeal cancers

Knowing the complex milieu of the oral microbial population, cross-talking with mucosal and immune cells, might be, therefore, the actual playground to understand the relationship between oral hygiene and cancer. If correct, the medical expert should be endowed with a thorough knowledge of which kind of oral microbiome is mostly represented in healthy individuals, respect to patients with oropharyngeal cancer. This investigation may start with comparing oral microenvironments with different degrees of daily hygiene.

Anderson and colleagues recently performed an RCT using stannous ions in the oral hygiene for three years compared with a control group and reported that the oral microbiome composition did not vary significantly between the two cohorts of subjects, but found that the genus *Prevotella* was particularly represented in the treated group, whereas the genus *Neisseria* and *Granulicatella*, were more abundant in the salivary samples of the control group (20).

Interestingly, despite the immune microenvironment the species *Porphyromonas gingivalis* has been recently associated with OSCC, despite many mechanisms by which this bacterial species promotes tumor progression is far to be fully elucidated (21). Several microbiota profiles reported that *P. gingivalis* in oral biofilms is associated with advanced stages and poor prognosis of esophageal squamous cell carcinoma (22). A possibility by which *P. gingivalis* activates the development of esophageal cancers should involve the NF-κB signaling (23). Actually, the causative and promotion role of *P. gingivalis* in oral cancers and in OSCC appears confirmed in recent reports and meta-analyses (24, 25). As *P. gingivalis* is the major causative microbial agent of periodontitis (26), therefore its association with oral cancers pertains to the direct association with previous gum and dental hygiene impairments leading to periodontitis (5, 27), and this may be beyond the purpose of our report.

Progressing with the role of the immune microenvironment, oral hygiene and the composition of the oral microbiome in the onset of pre-cancerous lesions and further of oral cancers, another study conducted on children reported that a relatively poor oral hygiene reduced the presence of the genus *Streptococcus* and increased the presence of the genus *Veillonella* (28). The interesting study performed by Gong and coworkers, reported that in patients with laryngeal squamous cell carcinoma (LSCC), swab specimens collected from the upper portion of the throat, near the epiglottis, reported the presence of the genus *Streptococcus* (38.8%), *Prevotella* (8.2%), *Veillonella* (7.3%), *Neisseria* (9.2%) and *Granulicatella* (2.8%) (29). Gong et al., reported that the genus *Prevotella* increased in LSCC patients, whereas the genus *Streptococcus* was significantly reduced (29).

However, Gong et al., concluded that the existence of a “pathological unit”, i.e. a complex multi-strain population of interacting genera and phyla, should be considered in any study relating oral microbiome with oropharyngeal cancers, instead of singular changes in the genus representation.

The highly stressed linkage between a scant oral hygiene and the etiopathogenesis of many oropharyngeal cancers, should be read not simply as the concurrent action of a pro-inflammatory micro-environment in triggering a malignancy but, most probably, in the upset homeostasis of the oral microbiome composition (30, 31).

This should suggest that the relationship between oral microbiome and cancers in the oral and pharyngeal tracts is still a crucial matter of debate. In this context, a possible question is: “Do dysbiosis modify the immune homeostasis in the oral cavity so to lead to a pro-carcinogenic microenvironment? or “Are particular bacterial species to be investigated as purported to be leading causative agents of oropharyngeal tumors”?

As indicated before, a possible straightforward, sound marker is *P. gigivalis*, or its presence in periodontits, as recently was reported by some authors for OSCC, where *P. gingivalis* was described as one of the leading causative bacterial strain of oral tumors (32). Gong and colleagues studied the relationship between oral microbiome and laryngeal carcinomas, concluding that impairments in the host’s oral microbiota structure and composition, may be major causative factors of laryngeal cancer (33). Most isolates in this case are saccharolytic and acid tolerant. *Streptococcus anginosus*, commonly linked with esophageal and oropharyngeal cancers, does not seem to be of significance in this kind of cancers. Similarly, significant salivary specificity is noted for three bacterial species, namely *Capnocytophaga gingivalis, P. melaninogenica*, and *Streptococcus mitis* in oropharyngeal cancer patients, making these species as major salivary markers for the early detection of these forms of cancers, thus improving the survival rate significantly (33, 34). Also, such high degree of bacterial specificity in oropharyngeal cancers has also prompted the design and testing of new treatment options for cancer prevention by way of vaccine delivery. However, for the success of these steps, a deeper exploration into this issue, ameliorating our previous knowledge, is warranted.

Sun et al., investigated the role of oral microbiota and associated specific species in the development of cancer, i.e. *Porphyromonas gingivalis, Fusobacterium nucleatum*, the order *Bacteriodales*, the genus *Latropia* and the species *Tannerella forsythia*, were associated with esophageal cancer and *Porphyromonas gingivalis*, the genus *Capnocytophaga, Dialisater, Filifactor, Catonella* and *Peptostreptococcus*, with oropharyngeal cancers (35). According to the authors, the genus *Streptococcus*, which encompasses a wide range (more than one hundred) of different strains and species, has controversial activities on promoting cancer, whereas streptococci in saliva were recently associated with gastric cancer (36), and *S. pneumoniae* was associated with lower risk of esophageal cancer (37). Investigating the immune interaction of specific bacteria with the oropharyngeal tissue and cell microenvironment, rather than bacterial species each other, probably represents a more suitable and affordable biomarker for cancer prevention and/or diagnosis. In this perspective, it would be particularly useful to investigate the physio-pathological state of those bacterial communities colonizing the oral cavity and the pharynx and their cross talk with the oral immune system.

From a microbiological point of view, it might be too simplistic, therefore, to ascribe the risk of oropharyngeal cancer to a single bacterial genus notoriously causing teeth and gum hygiene pathologies, because, as Gong and colleagues reported elsewhere (29, 33), the whole oral bacterial community and the interplays within, are to be focused to highlight possible causative factors for oropharyngeal tumors. This perspective should include, obviously, the impact of the microbial community on the immune homeostasis of the oral cavity.

Controversial issues in this context and study limitations are, yet, present.

Currently, oral microbiota in the oropharynx is still unexplored, even using culture-independent approaches and genomic research. Gong and colleagues recently characterized the composition and microbial structure of the pharynx in patients with laryngeal carcinoma, using a pyrosequencing of 16 sRNA libraries on 68 subjects with the carcinoma and 28 with only vocal cord polyps (2). *Firmicutes* represented the major phylum, whereas *Streptococcus* was the predominant genus, yet no difference between cases and controls in microbiota composition, as a whole, was observed (2).

In general, *Firmicutes* predominance was inversely related to *Fusobacteria*, *Actinobacteria*, *Proteobacteria* and *Bacteroidetes*, whereas the relative abundance of *Streptococcus* genus was inversely associated with the presence of *Prevotella*, *Actinomyces*, *Fusobacterium*, *Leptotrichia* and Neisseria (2). In a recent scoping review, performed on 274 papers of which only 9 eligible, authors reported that the association between oral tumors and oral microbiome was significant when changes in the microbiota composition occurred, particularly when an impaired abundance of the phyla *Firmicutes, Fusobacteria* and *Actinobacteria* and some species of the genus *Streptococcus, Actinomyces, Leptotrichia, Campylobacter* e *Fusobacterium* were predominant in the oral microbiome (38). In this study, tongue-related microbiome has been used as a predictor of cancers in the oral cavity (38). It would be interesting to assess the role of such microbiomes in tongue cancers, as well, as still representing a huge concern in oral oncology (39, 40).

The relationship between oral microbiome and oral cancers claims periodontal diseases as a major causative factor in oropharyngeal cancer, yet paucity in clinical and experimental reports cannot ensure about the reliability of this thesis, though intriguing (41).

## Does the immune microenvironment plays a role?

A first question oncologists should put forward regards the microbial composition in the nose-mouth-pharynx cavities, i.e. if an “optimal” bacterial population structure does exist and how to support and recover a balanced composition during oral dysbiosis events. Gut microbiome may be a possible specular model to elucidate the role of oral microbiome in cancer but differential biochemical microenvironments, either aerobic or anaerobic, in the oropharyngeal and gut microbiomes, might obviously be a matter of debate, due to those marked differences.

So, which kind of consideration one may achieve by looking into this evidence?

Gut microbiome dysbiosis has been recently associated with sporadic young-onset colorectal cancer (yCRC) and *Streptococcus* genus is again a major key phylotype in colorectal cancer, whereas yCRC is characterized also by the co-dominant presence of *Flavonifractor plautii* (42). The genus *Streptococcus*, in particular *S. anginosus*, has been linked with esophageal and pharyngeal cancers and interest in the involvement of oral microbiota in laryngeal cancers is recently increasing (3, 29, 43). Most of the bacterial isolates in the oral cavity are saccharolytic and acid tolerant species and are of poor significance in oropharyngeal cancer, including *Streptococcus anginosus*, which was controversially linked with esophageal and pharyngeal cancers (43). Yet, at least three bacterial strains, i.e. *Capnocytophaga gingivalis, P. melaninogenica*, and *Streptococcus mitis* in oropharyngeal cancer patients, were considered fundamental markers in salivary samples for early detection of oral tumors (43).

The role of *Streptococcus* genus may be particularly relevant, as with *Actinomyces* species, streptococci are the first oral colonizers anaerobic facultative bacteria. A possible homeostasis of the bacterial community in the oral cavity, may be ruled by most represented bacteria, such as some member of the *Streptococcus* genus (44).

Past reports appear quite intriguing in this sense, when streptococci were used to prevent cancer (45).

Oral streptococci modulate CD4^+^ CD25^+^ Foxp3^+^ regulatory T cells (T reg cells) *via* a TLR2-mediated NF-κB activation (46) and despite an exhaustive comprehension of the role of these cell subsets in oropharyngeal cancers is still far to be fully accomplished (47), some intriguing speculative hypothesis, can be put forward, at least on the basis of the unexpected and paradoxical protective role of some streptococcal strains on the cancer etiology in the oral microenvironment (48, 49).

T reg cells may be the key to understand how different species of commensal and resident bacteria in the oral cavities stay altogether in a homeostatic maintenance *via* the *Streptococcus* action on these CD4^+^ lymphocyte subsets. Actually, the oral mucosa very rarely experiences raw inflammatory events, despite the huge deal of micro-organisms occurring in its sites, an evidence suggesting that, somehow, immune activation is tolerated or attenuated. Hypotheses were forwarded about the possible role of Foxp3^+^ T regs cells in controlling the immune micro-environment in the oral mucosa. These cells are quite different as compared with spleen Foxp3^+^ Treg cells, as cells in the oral cavity express high amount of the membrane biomarker CD103 and high levels of CTLA4 (50), whose genetic polymorphism, i.e. particular polymorphic alleles, are associated with oral tumors (51). Actually, the acute depletion of these Treg cells cause severe pathology in oral mucosa.

One of the leading species modulating Foxp3 Treg cells in oral mucosa is *Streptococcus gordonii*, which usually disappears in laryngeal oral tumors to be replaced by other streptococcal species (33, 46). It is arguable that the *Streptococcus* genus, highly represented within the oral microbiome, may play a major role in maintaining the microbial homeostasis in the oral cavity by attenuating the immune response *via* the upregulation of Foxp3^+^ T reg cells (48). The participation of the *Streptococcus* genus to the aforementioned immune homeostasis occurs because this bacterial phylotype colonizes prevalently the oral mucosa as compared with other strains (51). Defined species, such as *S. mutans* and *S. subrirus*, causes caries, which has been associated with head and neck cancers, but not with laryngeal cancer (52).

Are there specific bacterial genus and species that act as preventive microbial barrier against a cancer onset? A possible response has to be searched in the complex field of the immune regulation, where Tregs and other immune cells participates in organizing an efficient cross talk between colonizing bacteria in the oral cavity and mucosal immunity. Despite this may appear as a speculative description of the role of oral microbiome in oropharyngeal cancer pathogenesis, further research should address the role of specific bacterial genus and Treg cells in this context.

Probably, science must address novel concepts in investigating the role of the oral microbial community as a microenvironment promoting cancer, such as the concept of oralome, which can be approached as the summary of the dynamic interactions orchestrated by a huge crowd of micro-organisms living in our own oral cavities (53).

## Conclusions

Does oral microbiome act as a friend or a foe in oral immunity? The search for some presumptive connection between oral bacteria, oral hygiene, lifestyle, voluptuary habits and predisposition to oropharyngeal tumors, is a burdensome task, as the relationship between a complex interacting microbiome with oral mucosal immunity and the gut microbiome is the actual playground of any sound research in this field. The mutual interplay between bacterial species present in the oral microbiome, particularly the genus *Streptococcus*, and the immune regulation by streptococcal-induced Foxp3^+^ Treg cells, should represent an insightful matter of debate to comprehend the onset and pathogenetic mechanisms of laryngeal oropharyngeal cancers. Insights on the pathophysiology of different oral microbiomes in patients with oropharyngeal cancers and on the interrelationship with the local oral mucosal immunity, may be the correct strategy to highlight and elucidate these issues. Oral microbiome, connected to lung and gut microbiome in a complex way, is able to modulate inflammatory signals *via* the orchestrated cross talks occurring between different species of resident bacteria, which promote, *via* their most represented strains, TLR-2 mediated activation of T cells, and maintain the oral microenvironment refractory to pro-inflammatory mechanisms leading to tumorigenesis. Further clinical studies in this sense may elucidate the great concern of oropharyngeal cancers. In particular, they may deepen the relationship with the oral microbiome, at least in order to focus on the factors enabling dentistry and head and neck medicine to set and recommend optimal preventive measures and successful therapies.

## Data availability statement

The original contributions presented in the study are included in the article/supplementary material. Further inquiries can be directed to the corresponding author.

## Author contributions

Conceptualization, RN, SC, NZ, MM and DG; methodology, DG, LM, MM, GM.; software, SC, NZ.; validation, NZ, DG, MM, GM; formal analysis, RN, SC, NZ.; investigation, SC, DG; resources, NZ, LM.; data curation, NZ, RN; writing—original draft preparation, SC, NZ, DG.; writing—review and editing, SC; visualization, RN, DG, NZ; supervision, RN, NZ, SC, DG. All authors have read and agreed to the published version of the manuscript.

## Conflict of interest

The authors declare that the research was conducted in the absence of any commercial or financial relationships that could be construed as a potential conflict of interest.

## Publisher’s note

All claims expressed in this article are solely those of the authors and do not necessarily represent those of their affiliated organizations, or those of the publisher, the editors and the reviewers. Any product that may be evaluated in this article, or claim that may be made by its manufacturer, is not guaranteed or endorsed by the publisher.
